# *QuickStats:* Percentage* of Children and Adolescents Aged 4–17 Years Who Practiced Yoga During the Past 12 Months,^†^ by Sex and Age Group — National Health Interview Survey,^§^ United States, 2022

**DOI:** 10.15585/mmwr.mm7246a6

**Published:** 2023-11-17

**Figure:**
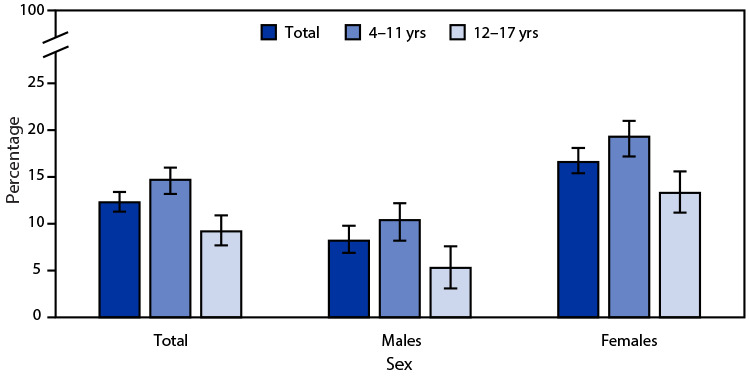
In 2022, 12.3% of children and adolescents aged 4–17 years had practiced yoga in the past 12 months. Children and adolescents aged 4–11 years were more likely to have practiced yoga than those aged 12–17 years (14.7% versus 9.2%). The declining percentages with age were found for both males and females: 10.4% versus 5.3% among males, and 19.3% versus 13.3% among females. Males were less likely than females to have practiced yoga in both age groups.

**Source:** National Center for Health Statistics, National Health Interview Survey, 2022. https://www.cdc.gov/nchs/nhis.htm

For more information on this topic, CDC recommends the following link: https://www.cdc.gov/healthyschools/bam/cards/yoga.html

